# Rapid bursts and long-term stasis: phylogeny, evolutionary dynamics, and expression of B3 transcription factors in *Arabidopsis*, *Cannabis*, and *Humulus*

**DOI:** 10.3389/fpls.2026.1882604

**Published:** 2026-10-15

**Authors:** Matteo Toscani, Ainhoa Riera-Begue, Nina Trubanová, Susanne Schilling, Rainer Melzer

**Affiliations:** School of Biology and Environmental Science and Earth Institute, University College Dublin, Dublin, Ireland

**Keywords:** B3, Cannabis sativa, Humulus lupulus, REM, *REM16*, B3 transcription factor

## Abstract

The B3 superfamily is one of the largest plant transcription factor gene families, yet its evolutionary dynamics in dioecious species like *Cannabis sativa* remain largely unexplored. Here, we present a comprehensive phylogenetic, molecular evolution, and transcriptomic analysis of B3 domain-containing genes in *Arabidopsis thaliana*, *C. sativa*, and *Humulus lupulus*. Using a gene mining pipeline, we identified 110, 101, and 88 B3 genes across the five established subfamilies (*ARF*, *ABI3*, *HSI*, *RAV*, and REM), respectively. We identified two distinct evolutionary trajectories with the B3 superfamily. On the one hand the REM subfamily was by far the largest and characterized by massive lineage-specific tandem duplications subjected to relaxed selective constraint, suggesting distinct or partially redundant roles in reproductive development. On the other hand, specific non-tandem clades remained highly conserved under strong purifying selection. Detailed analysis of the candidate sex-determination gene *CsREM16* showed low dN/dS ratios, clear one-to-one orthology across studied species, and unique persistent sex-biased expression across all six analyzed developmental stages in *C. sativa*. Comparative analysis of its ortholog *HlREM16* confirmed a conserved female-biased expression in *H. lupulus*. Together, these results demonstrate a dual-mode evolutionary dynamic in the B3 superfamily and particularly in the REM genes and establish *CsREM16* as a strong candidate for sex determination in *C. sativa*, with a potentially conserved role in *H. lupulus*.

## 1 Introduction

*Cannabis sativa* (hemp) and its close relative *Humulus lupulus* (hop) are dioecious species within the Cannabaceae family, with sex determined mainly by an XY chromosome system in which females are XX and males are XY. The genetic mechanisms governing sex expression in hemp have long remained elusive, but recent transcriptomic approaches have begun to identify key candidate genes. Through differential gene expression and co-expression network analysis of male and female plants across developmental stages, it was previously found that sex-biased gene expression is established very early in the vegetative phase, well before reproductive development begins (Shi et al., 2025). Moreover, several transcription factor genes were identified as candidate sex determinants, among them *CsREM16*, an X-linked homolog of the *Arabidopsis thaliana* gene REPRODUCTIVE MERISTEM16 (*REM16*), which belongs to a gene family implicated in reproductive development across plant species. REM genes belong to the B3 superfamily, one of the largest and most ancient plant-specific transcription factor families (Swaminathan et al., 2008).

The B3 superfamily comprises several families: the auxin response factors (ARF), the LEAFY COTYLEDON 2/ABSCISIC ACID INSENSITIVE 3 (LEC2/ABI3) and HIGH-LEVEL EXPRESSION OF SUGAR-INDUCIBLE GENE/VP1/ABI3-LIKE (HSI/VAL) families, the Related to ABI3/VP1 (RAV) family, and the *REPRODUCTIVE MERISTEM* (REM) family (Romanel et al., 2009). In recent years, B3 transcription factors have been characterized in a number of species (Wei et al., 2023; Wang et al., 2022; Wu et al., 2026; Lu et al., 2024; Wang et al., 2024). In general, B3 transcription factors play important roles in plant growth, seed development, and hormonal signaling. VAL-, ABI3-, and RAV-like genes constitute comparatively small subfamilies and are relatively well characterized, at least in *A. thaliana* (Wang et al., 2012; Cheng et al., 2026; Boulard et al., 2017). ARF- and REM-like genes consistently form the largest and most diverse B3 subfamilies (Wang et al., 2012). ARF genes are important players in auxin-triggered gene expression changes (Hernández-García et al., 2024; Li et al., 2016).

In contrast to the above-mentioned subfamilies, the *REM* subfamily in particular remains relatively poorly characterized despite (or maybe because of) its significant expansion across plant genomes (Park et al., 2024; Wang et al., 2022; Zameer et al., 2025). Despite this overall lack of functional characterization, the few functionally studied REM genes highlight their crucial, specialized roles in reproductive biology. In *A. thaliana*, the REM transcription factors VERDANDI (VDD) and VALKYRIE (VAL) are directly downstream of the MADS-domain protein complex SEEDSTICK-SEPALLATA3 and interact to form a complex that controls female gametophyte development, in particular synergid cell death, which is essential for successful fertilization (Mendes et al., 2016). Additionally, other closely related REM genes (such as *REM34* and *REM35*) have been shown to be required for the proper development of both male and female gametophytes (Caselli et al., 2019). Beyond their direct roles in gametophyte development, REM genes have recently been shown to play important roles in shaping epigenetic regulation in plant reproductive tissues. In *A. thaliana*, several REM genes encode proteins that recognize specific DNA sequence motifs and, together with accessory proteins, recruit the RNA polymerase IV complex to direct the production of small interfering RNAs (siRNAs) and subsequent DNA methylation at specific loci in both ovules and anthers (Xu et al., 2025; Wu et al., 2025). Furthermore, REM genes have been associated with controlling flowering time, phyllotaxy, and cell cycle progression (Manrique et al., 2023; Ferrario et al., 2026). Because REM genes are intrinsically connected to the pathways governing reproductive development, male and female gamete formation, and floral organogenesis, they represent some prime evolutionary candidates to be co-opted or rewired as master regulators of sex determination in dioecious species. Yet, the evolutionary dynamics and functional diversification of B3 genes, in particular the REM subfamily, have not been systematically investigated to date in dioecious crops like hemp.

Here, we characterize the B3 gene family across *C. sativa*, *H. lupulus*, and *A. thaliana* through phylogenetic and molecular evolution analysis, and transcriptomics, with particular focus on the REM subfamily and the candidate sex-determination gene *CsREM16* (Shi et al., 2025; Toscani et al., 2026). Notably, our evolutionary analyses reveal that *CsREM16* is evolving under extremely strong purifying selection, suggesting an evolutionary trajectory and functional divergence potentially linked to the evolution of sex chromosomes in *Cannabaceae*.

## 2 Materials and methods

### 2.1 Plant growth and sample collection

Seeds of the dioecious cultivar ‘FINOLA’ (photoperiod-insensitive) were sown in a mixture of compost (John Innes no. 2), vermiculite, and perlite (2:1:1). After 2 days of incubation in the dark, plants were grown under an 18:6 photoperiod (red-blue LEDs) in a controlled growth room at 22–25 °C.

Samples were collected at three developmental stages: germination, cotyledon, and first-true leaf stage (L1); a total of 15 biological samples were collected per stage.

Stages were defined as follows: germination stage is the point at which the radicle is already out, but the seed coat is still on. For sampling, the seed coat was removed, and the naked seed was used for RNA isolation, and the radicle for DNA isolation and genotyping. Cotyledon stage refers to when cotyledons have clearly emerged, but no true leaf is visible. Both cotyledons are collected, one for RNA isolation and the other one for DNA isolation and sex genotyping. The L1 stage is when the first-true leaf is completely developed and the second-true leaf is a maximum of 5 mm. Apical structures including newly emerged leaves up to 5 mm in length were harvested for RNA isolation, and a leaf for DNA isolation and genotyping.

At these early stages, it was not possible to differentiate male and female plants, so sex genotyping was performed using the CsPDS5-CAPS protocol (Riera-Begue et al., 2025). After determining sex, five female and five male individuals per developmental stage were selected for RNA isolation and sequencing.

### 2.2 Mining of B3 domain gene family members

B3 domain-containing genes were identified across three plant genomes using the GENE-FAM pipeline (https://github.com/Louise-Ryan/GENE-FAM). The genomes analyzed were *A. thaliana* (GCF_000001735.4, TAIR10.1) (Lamesch et al., 2012), *C. sativa* (GCF_029168945.1, ASM2916894v1) (Ryu et al., 2024), and *H. lupulus* (GCF_963169125.1, drHumLupu1.1) (Christenhusz et al., 2025). The corresponding RefSeq genome assembly, protein annotation, transcript, coding sequence, and gene annotation files were downloaded from the NCBI RefSeq FTP server.

Protein and nucleotide HMM profiles were built using HMMER v3.4 (Eddy, 2011) with the Pfam PF02362.26 B3 domain seed alignment and a nucleotide alignment of 64 *A. thaliana* B3 domain CDS sequences. Each genome was searched using hmmsearch against the protein HMM and nhmmer against the transcript and CDS sequences (E-value ≤0.01). Hits from all searches were retained, with gene coordinates and locus identifiers obtained from the GFF annotation. Where multiple transcript isoforms existed for a given locus, the isoform with the longest CDS was selected.

To identify unannotated gene family members, nhmmer was additionally run against the whole genome assembly of each species. Non-overlapping genomic hits were extracted with flanking sequence and gene models predicted using AUGUSTUS v3.5.0 (Stanke et al., 2006) with the *A. thaliana* species model. Predicted genes were retained only if they overlapped at least 90% of the nhmmer-identified domain region. Predictions passing this overlap criterion were further validated by running hmmsearch on the predicted protein sequence against the B3 protein HMM profile. Genes were classified as pseudogenes if they contained premature stop codons or were <300 nt in length, and the pipeline identified putative duplicates via an all-versus-all BLASTp run.

Genes lacking protein-level HMM support were excluded from subsequent phylogenetic analyses.

### 2.3 Alignment, trimming, and phylogenetic tree building

Full-length B3 domain-containing protein sequences identified in *C. sativa*, *H. lupulus*, and *A. thaliana* were aligned using the MAFFT v7 online tool (Katoh and Standley, 2013) with default parameters. The resulting multiple sequence alignment was trimmed with trimAl v1.5 using a gap threshold of 0.7 (-gt 0.7, at least 70% of rows have to be non-gaps) to remove poorly aligned and gap-rich columns, yielding a final alignment of 298 sequences spanning 110 positions.

Maximum likelihood phylogenetic reconstruction was performed using IQ-TREE3 v3.0.1 (Wong et al., 2026). The best-fit substitution model was selected automatically via the ModelFinder integrated within IQ-TREE, which ran JTT + F + R4 as the optimal model according to the Bayesian Information Criterion (BIC), and branch support was assessed using the ultrafast bootstrap approximation UFBoot (Hoang et al., 2018) with a 2,000-replicate parameter (-B 2,000).

### 2.4 B3 family assignment

To assign each identified *C. sativa* and *H. lupulus* B3 gene to its respective B3 family clade, the Romanel classification of *A. thaliana* B3 superfamily genes (Romanel et al., 2009) was used as a reference.

First, each known *A. thaliana* B3 gene ID was assigned to the corresponding established B3 family classification. Then, each non-*Arabidopsis* gene (*H. lupulus* and *C. sativa*) was assigned to the subfamily of its topologically closest *A. thaliana* gene in the tree. To do that, for each non-*Arabidopsis* gene, pairwise most-recent common ancestors (MRCAs) with all *A. thaliana* genes were found using the APE R package (Paradis et al., 2004), and the *A. thaliana* gene whose MRCA subtended the fewest descendant tips was selected as the nearest reference. The gene was then assigned to that *A. thaliana* reference gene’s B3 family. Once each gene was assigned to a corresponding B3 family, genes were named systematically using sequential numbering within each family, ordered by their position in the alignment.

### 2.5 Evolutionary rates analysis

Two focal datasets were extracted from the B3 superfamily phylogeny for molecular evolution analyses:

First, the *REM16* clade formed a monophyletic clade that includes *CsREM16* (LOC115699937), the *Cannabis* ortholog of *A. thaliana REM6/REM16* (AT4G33280). After aligning and trimming with a -gt 0.7 threshold, eight genes with >30% gaps were removed, leaving in the clade 41 genes (28 *A. thaliana*, six *C. sativa*, seven *H. lupulus*).

Then, the *C. sativa* chromosome 4 REM tandem cluster comprised a tandem B3 gene array on *C. sativa* chromosome 4 and their *H. lupulus* orthologs. After 34 gappy sequences (>30% gaps after initial alignment trimming) were removed, the clade was composed of 35 genes (20 *C. sativa*, 15 *H. lupulus*), and the remaining sequences re-aligned.

In both datasets, AT5G42700 (*REM 2* from *A. thaliana*), a *REM* gene outside both clades, was included as an outgroup for tree rooting, yielding final datasets of 42 and 36 sequences, respectively.

Sequences retaining a high proportion of gaps can bias branch-length and ω estimates and increase false-positive rates in codon-based maximum-likelihood tests of selection (Markova-Raina and Petrov, 2011). To mitigate this, the stringent limit of <30% gaps after initial alignment was applied.

After removing the gappy genes, the protein sequences were aligned with MAFFT, and codon alignments were generated by aligning CDS sequences onto the protein alignment using PAL2NAL v14 (Suyama et al., 2006), with a gap-threshold of 0.7 for both datasets, retaining the alignment columns present in at least 70% of sequences.

Maximum-likelihood gene trees for both datasets were inferred from the trimmed protein alignments using IQ-TREE 3.0.1, with ModelFinder used for model selection. Selection pressures were estimated using codeml in PAML 4.10.10 (Yang, 2007) with FMutSel0 (CodonFreq = 7, estFreq = 1), treating gaps as ambiguity data (cleandata = 0).

The following models were fitted:

A free-ratio model (model = 1, NSsites = 0) was used to estimate branch-specific dN/dS (ω) values in both datasets.

A two-ratio branch model was then applied to the 12 chromosome 4 cluster terminal branches with free-ratio ω <0.5, compared with a one-ratio null model using a likelihood-ratio test (LRT, 1 df).

A two-ratio branch model was also used for the *CsREM16* terminal branch, tested against neutrality (ω = 1; LRT, 1 df). In addition, a branch model with the *Cannabis–Humulus REM16* ancestor branch as foreground, as in the previous model, labeled to test whether its ω rate deviates significantly from neutrality.

Finally, a branch-site model (model = 2, NSsites = 2) was fitted for the Cannabis–Humulus *REM16* ancestral branch, comparing H1 (ω_2_ >1) with H0 (ω_2_ = 1; LRT, 1 df). This model tried to identify positively selected sites in the branch with a Bayes Empirical Bayes analysis.

The tip branch omega values (excluding branches with ω >10, which reflect near-zero synonymous divergence rather than true dN/dS signal) were compared between the *REM16* clade and Chr4 cluster clade using a two-sided Wilcoxon rank-sum test.

### 2.6 Collinearity analysis of the REM arrays

To examine the evolutionary relationships between the putative *REM* tandem repeat arrays, we used the B3 protein phylogeny tree and focused on the 36 REM genes in the *C. sativa* chromosome 4 array and the 19 REM genes in the *H. lupulus* chromosome 1 array. The orthology relationships were inferred from the topology of the complete gene tree using a species-overlap rule. For each internal node, we compared the species represented in its two descendant branches. A node was interpreted as a speciation node when the two branches contained both species. If the same species occurred in both branches, the node was interpreted as a gene-duplication node. A *Cannabis–Humulus* gene pair was considered a clear orthology relationship only when its most recent common ancestor was a speciation node. When duplication after speciation produced several genes in one or both lineages, all supported co-orthologs were retained.

The inferred relationships were displayed together with the physical positions of the genes in the two arrays. Genes were ordered by their genomic midpoints along *C. sativa* chromosome 4 and *H. lupulus* chromosome 1. Connecting lines indicate clear orthology or co-orthology relationships supported by a speciation node in the gene tree.

### 2.7 RNA isolation and sequencing

The RNA was isolated using the RNeasy^®^ Plant Mini Kit (Qiagen, Germany) according to the manufacturer’s instructions. The total number of samples was 30; RNA integrity number (RIN) and concentration were determined using the Qubit RNA IQ Assay Kit (Invitrogen, USA) and were >7 and >10 ng/μL, respectively.

RNA sequencing (RNAseq) was performed using a strand-specific protocol (polyA library prep mRNA enrichment, Illumina NovaSeq X Plus, PE150 sequencing, 8 GB data output per sample; Novogene (UK) Company Limited). Sequencing quality was high, with ≥85% of bases achieving a Q30 score.

### 2.8 Differential expression analysis

RNA-seq paired reads were mapped to the reference transcriptome (https://www.ncbi.nlm.nih.gov/datasets/genome/GCF_029168945.1/) using Salmon (version 1.10.3) (Patro et al., 2017). Since the reference transcriptome is from a female plant, the Y transcripts from a Salk male genome were appended (https://resources.michael.salk.edu/root/tools.html?tool=%2Fresources%2Fcannabis_genomes%2Findex.html).

Transcript-level abundances generated with Salmon were imported into R (version 4.4.0) using the tximport package (Soneson et al., 2015), which summarized transcript abundances to gene-level counts based on a custom transcript-to-gene mapping derived from the reference genome annotation, including Y-chromosome-specific annotations.

All differential gene expression analyses were conducted using DESeq2 (version 1.44.0) (Love et al., 2014). Across all analyses, genes with low expression were filtered out by retaining only genes with more than 10 raw counts in at least three samples. Statistical significance was assessed using an adjusted p-value <0.05 and an absolute log2 fold change >1.

## 3 Results

### 3.1 Identification of the B3 domain-containing genes across *Cannabis*, *Humulus*, and *Arabidopsis*

Using the GENE-FAM pipeline (https://github.com/Louise-Ryan/GENE-FAM) (Ryan et al., 2026), we identified and validated B3-domain-containing genes in the three reference genomes of *A. thaliana*, *C. sativa*, and *H. lupulus*, identifying a total of 299 non-redundant loci retained across the three species that passed the GENE-FAM HMM protein composition filter step and were classified as functional genes (no B3 transcription factor was flagged as a pseudogene in any of the three genomes).

In *A. thaliana*, 110 B3 gene loci were identified (**Figure 1**; **Supplementary Table 1**), 26 of which were not previously identified when characterizing the B3 superfamily (Romanel et al., 2009). The 110 genes were relatively evenly distributed across all five chromosomes.

**Figure 1:**
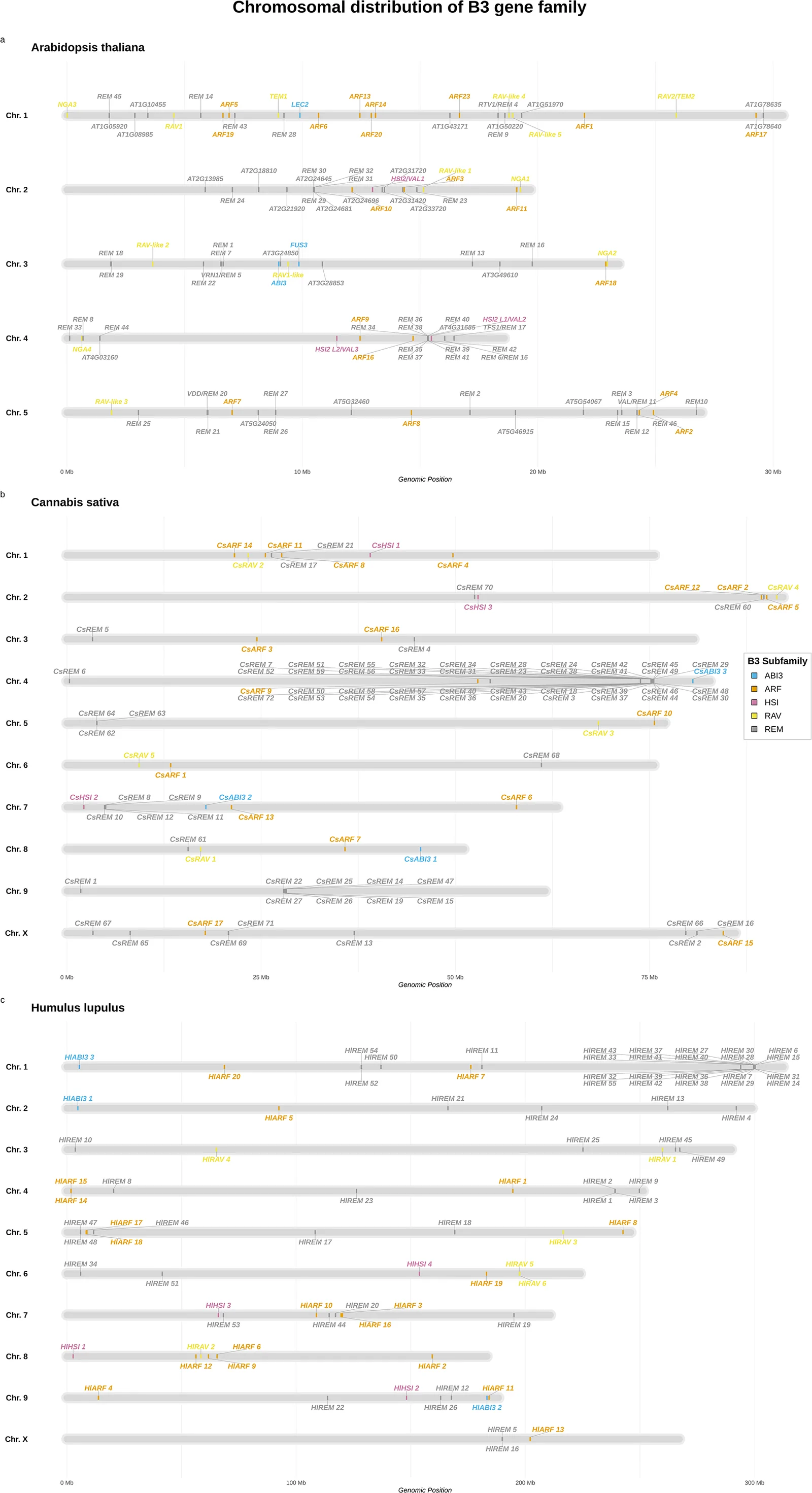
Chromosomal distribution of the B3 superfamily genes in the *A. thaliana*, *C. sativa*, and *H. lupulus* genomes. Karyogram detailing the physical mapping position of identified B3 genes across the **(A)** *A. thaliana*, **(B)** *C. sativa*, and **(C)** *H. lupulus* genomes. Horizontal gray bars are the individual chromosomes, with the x-axis indicating the genomic position in megabases (Mb). The exact genomic locations (midpoints) of the B3 genes are indicated by colored vertical tick marks, with lines connecting them to their respective gene names. Both the gene names and their corresponding tick marks are color-coded to denote their specific B3 gene family clade: ARF (orange), ABI3 (sky blue), HSI (reddish purple), RAV (yellow), and REM (gray).

A total of 101 B3 domain-containing genes were identified in *C. sativa* (**Supplementary Table 1**). A notable feature of the *C. sativa* B3 genes’ chromosomal position is the pronounced clustering on a small section of chromosome 4 (NC_083604.1), which alone harbored 36 of the 72 REM genes identified in *C. sativa* (50%), with 33 of these (92%) located on the forward strand. The remaining genes were more evenly spread across the other nine chromosomes (3–10 genes each) (**Figure 1**).

In *H. lupulus*, 88 B3 gene loci were identified (**Supplementary Table 1**). Genes were distributed across the 10 *H. lupulus* chromosomes (**Figure 1**), with counts ranging from 3 to 27 per chromosome and the largest cluster was found on chromosome 1 (NC_084793.1), containing 19 REM genes, approximately 35% of all identified REM genes in *H. lupulus*, with 18 of those (95%) being located on the forward strand.

### 3.2 Phylogeny reconstruction and subfamily assignment

After aligning the protein sequences of the identified B3 genes and trimming the alignment to retain sites present in at least 70% of sequences, a total of 298 B3 domain-containing genes were retained for phylogenetic analysis (one gene was excluded as it missed all of the retained post-trimming sites). Maximum-likelihood phylogeny reconstruction using IQ-TREE 3 allowed us to recover the five clades corresponding to the previously described B3 subfamilies: ARF (56 genes), ABI3 (9 genes), HSI (10 genes), RAV (24 genes), and REM (199 genes) (**Table 1**; **Figure 2**). The REM subfamily was by far the largest, comprising 66.8% of all B3 genes in the dataset. Species representation within each clade was broadly consistent across the three genomes (**Table 1**; **Supplementary Table 1**), with the REM clade having slightly more variation between species: *C. sativa* and *A. thaliana* each contained 72 genes (72% of all the B3 genes in *C. sativa* and 65.5% in *A. thaliana*), while *H. lupulus* had 55 (62.5% of all *H. lupulus* B3 genes).

**Table 1:** B3 gene families summary.

Species	ABI3	ARF	HSI	RAV	REM	Total B3 genes
*A. thaliana*	3 (2.7%)	19 (17.3%)	3 (2.7%)	13 (11.8%)	72 (65.5%)	110
*C. sativa*	3 (3%)	17 (17%)	3 (3%)	5 (5%)	72 (72%)	100
*H. lupulus*	3 (3.4%)	20 (22.7%)	4 (4.5%)	6 (6.8%)	55 (62.5%)	88

**Figure 2:**
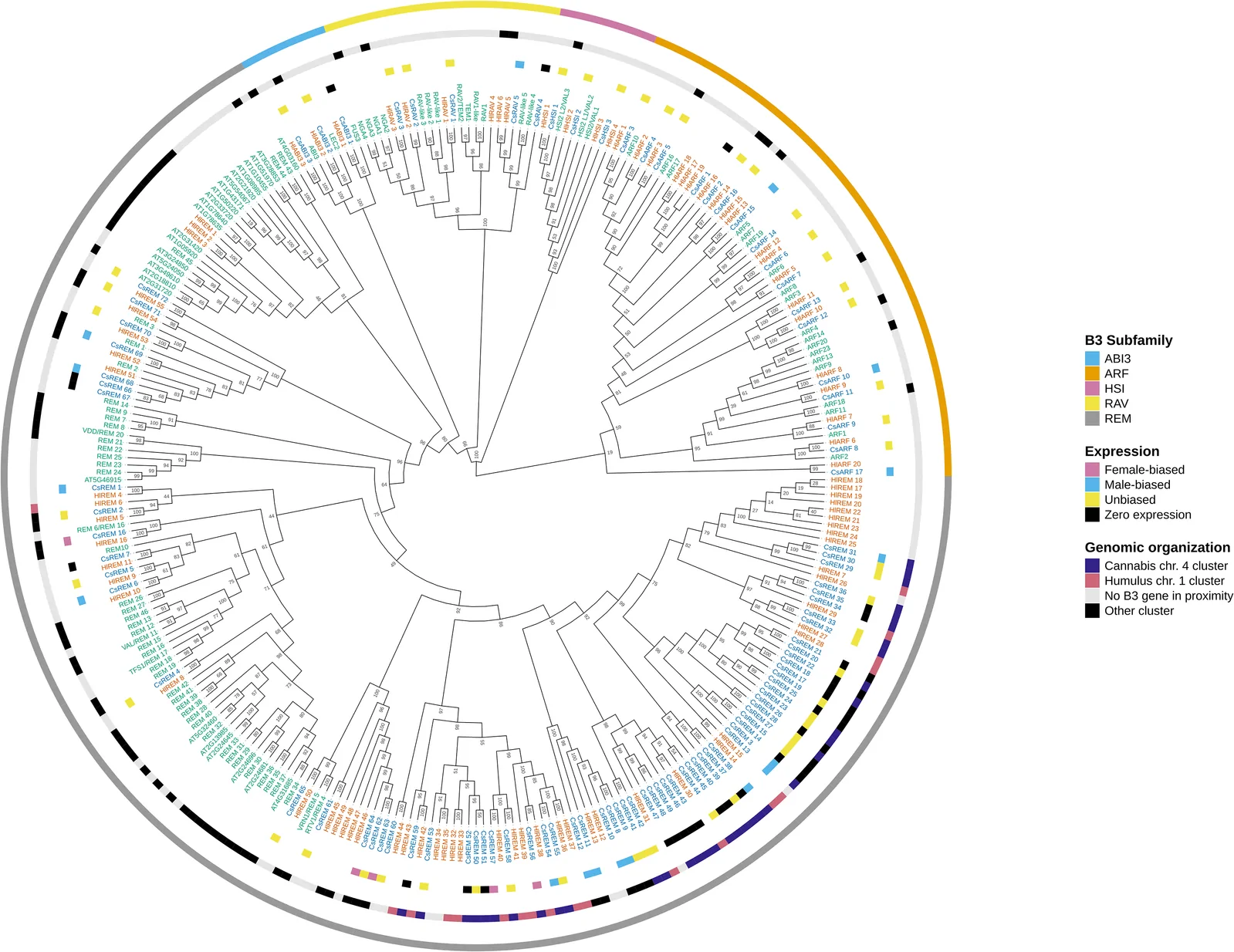
Maximum-likelihood phylogeny, subfamily organization, and expression profiles of the B3 gene superfamily. A phylogenetic tree of the 298 B3 domain-containing genes mined from *A. thaliana*, *C*. *sativa*, and *H*. *lupulus* genomes. The tree topology was inferred using maximum-likelihood reconstruction (branch lengths are here omitted for visual clarity). The outermost strip indicates the classification into five recovered B3 gene families, with subfamily assigned based on the closest *A. thaliana* homolog: ARF (56 genes, orange), ABI3 (9 genes, sky blue), HSI (10 genes, reddish-purple), RAV (24 genes, yellow), and REM (199 genes, gray). The middle strip denotes genomic organization in putative tandem duplications, defined as the presence of another B3 gene within a 100 kb genomic window. Genes located within the two largest putative tandem arrays are specifically highlighted: the *C. sativa* chromosome 4 cluster (dark indigo) and the *H. lupulus* chromosome 1 cluster (rose). Other tandemly duplicated genes are marked in black, while non-tandem genes are indicated by light gray. The innermost strip displays sex-biased expression patterns in *C. sativa* apical meristems, based on the transcriptomic data described above, categorized as female-biased (dark orange), male-biased (blue), or unbiased (green). Genes are characterized as male- or female-biased if they were found to be sex-biased at any of the stages examined.

Notably, the topology of the phylogenetic tree revealed distinct evolutionary patterns between the B3 subfamilies. For the ABI3, ARF, HSI, and RAV clades, gene phylogenies followed species phylogenies in many cases, and clear orthologs between *C. sativa*, *H. lupulus,* and *A. thaliana* could be identified (**Figure 2**). In some cases, gene duplication specific to one species could be detected (for example, *ARF7* and *ARF19* in *A. thaliana*). In stark contrast, in the REM subfamily, large clades with genes from just one species or with genes only from *H. lupulus* and *C. sativa* were detected, and the assignment of clear orthologs across all three species was often uncertain. To confirm this and improve the phylogenetic resolution, an analysis was performed on the 199 REM genes alone. This yielded a tree that showed essentially the same pattern of species-specific expansion of clades (**Supplementary Figure 1**). Evolutionary relationships of functionally characterized genes within the REM subfamily present a mixed pattern of conservation. Certain functionally characterized *A. thaliana genes*, like *VAL/REM11* (Mendes et al., 2016), entirely lack clear orthologs in *C. sativa* and *H. lupulus*. In contrast, genes such as *VRN1/REM5* (King et al., 2013) and *RTV1/REM4* (Heo et al., 2012) cluster closely with *C. sativa* and *H. lupulus* genes. Notably, these relationships are not strictly one-to-one, but instead, the *C. sativa* and *H. lupulus* genes, *CsREM65* and *HlREM*50, appear as co-orthologs to both *VRN1* and *RTV1*.

Our previously identified sex-determination candidate gene *CsREM16* from *C. sativa* (Toscani et al., 2026) showed a different pattern: in contrast to the widespread lineage-specific duplications observed across the overall REM family clade, the *REM16* subclade exhibited a remarkably conserved phylogenetic pattern. *CsREM16* (LOC115699937) showed a clear 1:1:1 orthology to *A. thaliana REM6/REM16* (AT4G33280) and *HlREM16* (LOC133803719), forming a well-supported clade (UFBoot = 100%).

A striking feature of the REM clade is the prevalence of REM genes located closely together in the genome: 128 of 199 REM genes (64.3%) were located within 100 kb of another B3 family member on the same chromosome, compared to only 14 of 99 non-REM B3 genes (14.1%) (**Supplementary Table 1**). Those putative tandem duplications were particularly prominent in *C. sativa* (**Figures 1**, **2**), where 57 of 72 REM genes (79.2%) occurred in close proximity to other B3 genes, the largest of which was a dense cluster of 36 REM genes spanning only ≈350 kb on chromosome 4 (75.19–75.54 Mb). *H. lupulus* and *A. thaliana* REM genes also showed substantial, albeit lower, putative tandem repeat frequencies (28/55, 50.9% and 43/72, 59.7% of REM genes, respectively).

Phylogenetic analysis of the genes from the putative tandem repeat clusters (**Figure 1**, **2**) identified *C. sativa* orthologs for 15 of the 19 REM genes in the *H. lupulus* chromosome 1 cluster. Further analyses demonstrated synteny between *H. lupulus* cluster 1 and *C. sativa* cluster 4 genes and also showed that several co-orthologous groups likely originated by lineage-specific duplications (**Supplementary Figure 2**).

Analysis of exon–intron structure and B3 domain composition across the five B3 subfamilies (**Supplementary Table 2**; **Supplementary Figure 3**) revealed some clade-specific characteristics. The ARF and HSI clades displayed the gene structures with the highest exon number, with median exon counts of 13 per gene. The ABI3 clade showed lower complexity (median: 6 exons). In contrast, the RAV clade exhibited a markedly lower exon number with a median of 2 exons per gene. The REM clade showed a moderate exon number with a median of 5 and the presence of multiple B3 domains (**Supplementary Table 2**). While the ARF, HSI, and RAV clades consistently harbored a single B3 domain per gene, and ABI3 members showed near-uniform single-B3 domain composition, the majority of REM genes contained two B3 domains (**Supplementary Figure 3**).

### 3.3 Evolutionary rates analysis

To characterize the selective pressures acting on the putative sex-determination candidate *CsREM16* and closely related genes, we focused on a REM clade containing *CsREM16* and compared it to a clade containing a cluster of REM genes tightly positioned on chromosome 4, possibly originating from tandem duplications. To compare the evolutionary dynamics of those clades, PAML free-ratio models, which infer the best-fitting dN/dS (here referred to as omega, *ω*) value for each branch of the tree, were fitted to each dataset independently (**Figure 3**). In the clade containing *REM16*, most genes showed ω values well below 1 (**Figure 3A**). In contrast, the Chr4 tandem cluster exhibited predominantly higher ω values on terminal branches (**Figure 3B**). Excluding terminal branches with ω >10, the median ω for terminal branches in the clade containing *REM16* was 0.391 (n = 36 branches) compared to 0.590 for the Chr4 cluster (n = 35 branches). A Wilcoxon rank-sum test confirmed that Chr4 cluster genes have significantly higher ω than *REM16* clade genes (W = 963, p = 8.4 × 10^−5^).

**Figure 3:**
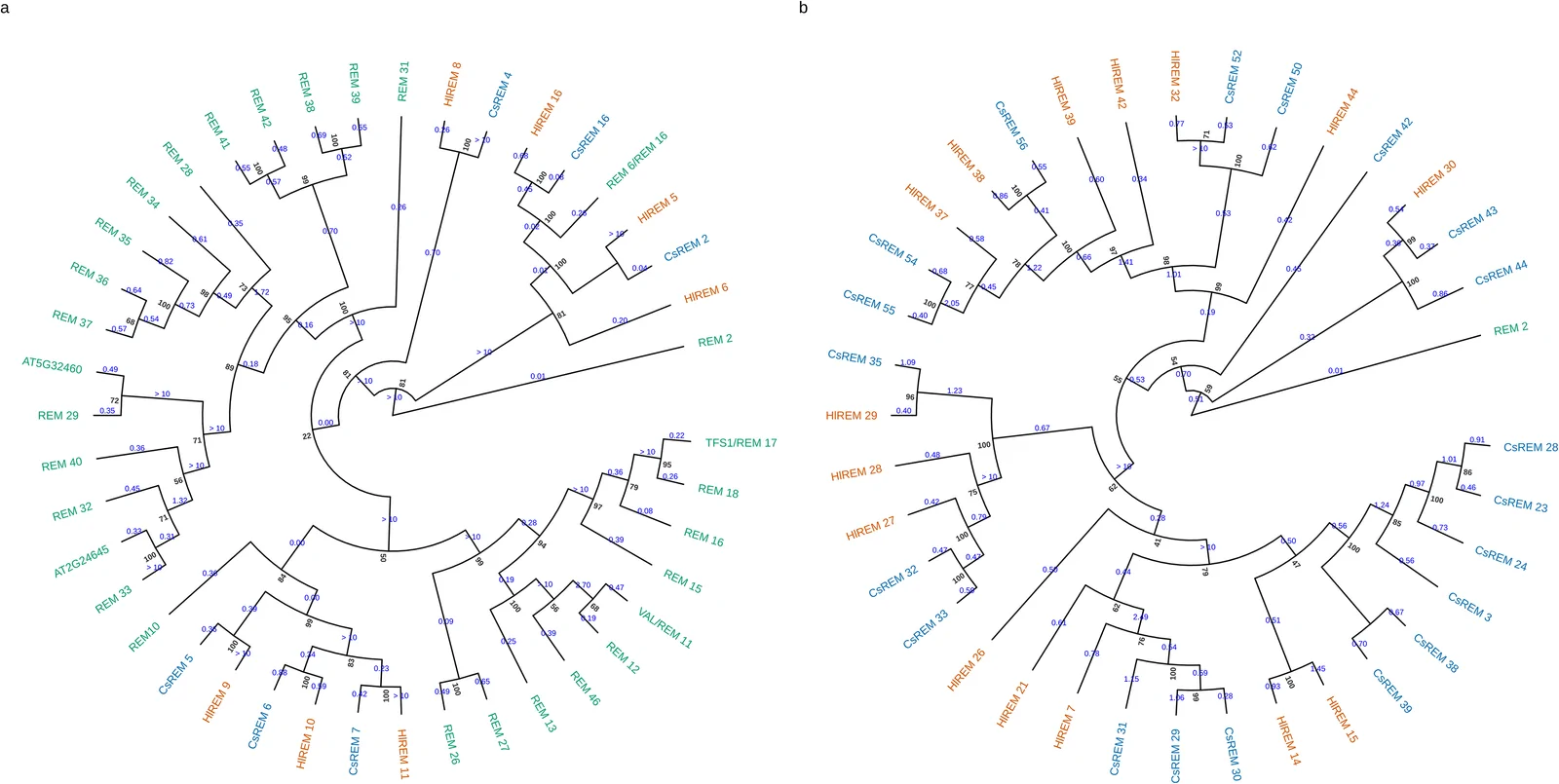
Trees and selection pressure analysis of the *REM16* clade and Chr4 cluster. Maximum likelihood phylogenetic circular trees for the **(A)** *REM16* clade and **(B)** Chr4 REM cluster. Dark gray labels at internal nodes indicate IQ-TREE3 UltraFast Bootstrap support percentages (2,000 iterations). Blue value labels positioned along the branches represent the ratio of nonsynonymous to synonymous substitution rates (ω = dN/dS). The ω values were estimated using the PAML codeml branch-specific free-ratio model (model = 1, NSsites = 0) with the FMutSel0 mutation-selection model enabled for codon frequencies (CodonFreq = 7). To facilitate visualization, branches with highly elevated ω estimates are displayed as “>10” and branch lengths are ignored when plotting.

To further confirm that the clade containing the Chr4 REM cluster is predominantly characterized by relaxed or neutral evolution, we isolated the minority of genes in the clade exhibiting signatures of strong purifying selection (defined here as ω <0.5). Based on the free-ratio model, only 12 terminal branches met this threshold: six of the 20 (30%) *C. sativa* genes and six out of 16 (37%) *H. lupulus* genes, whereas for the clade containing *REM16*, 26 out of 42 (62%) met this threshold (**Figures 3A, B**). A two-ratio branch model with all 12 low-ω terminal branches in the Chr4 REM clade jointly labeled as foreground confirmed that these branches evolved under significantly stronger purifying selection than the clade average (foreground ω = 0.403; LRT = 35.11, df = 1, p = 3.1 × 10^−9^; **Table 2**). All the remaining genes showed ω values consistent with relaxed, closer-to-neutral evolution (ω ≥0.5).

**Table 2:** Evolution rates analysis on chr 4 putative tandem repeats cluster.

PAML test	Foreground branches	Dataset	Model	lnL_H0	lnL_H1	n parameters H0	n parameters H1	LRT	df	P_value	ω background	ω foreground branches
Branch model	12 genes with ω <0.5	36 genes	two-ratio	−25,850.14	−25,832.58	134	135	35.11292	1	3.11E−09	0.664	0.403

PAML codeml two ratio model run tagging low ds genes in the clade containing the chr. 4 putative tandem repeats cluster.

Furthermore, we hypothesized that the low median ω in the clade containing *CsREM16* is primarily driven by *CsREM16* and its most closely associated genes, which appear to be under stringent purifying selection with ω values approaching zero. To test this, we implemented a PAML branch model on the overall *CsREM16-*containing clade, in which we designated *CsREM16* and its five closest relatives as foreground branches (alternative hypothesis, H1), allowing their ω to vary independently from the rest of the tree (background), which was compared against a null model (H0) assuming a uniform ω. A likelihood ratio test (LRT) revealed a significantly better fit for the H1 model (LRT = 28.44, df = 1, *p* = 9.65 × 10^−8^, **Table 3**). As predicted, the foreground branches exhibited a significantly lower ω (0.185) compared to the background branches (0.429).

**Table 3:** Evolution rates analysis on clade containing *REM16*.

PAML test	Foreground_branch	Dataset	Model	lnL H0	lnL H1	LRT	n parameters H0	n parameters H1	df	p value	ω background	ω foreground branch
Branch model	*CsREM16*	42 genes 245 codons	two-ratio	−25,958.56	−25,950.18	16.76546	146	147	1	0.0000423	0.411	0.094
Branch model	*HlREM16*	42 genes 245 codons	two-ratio	−25,957.27	−25,952.11	10.300546	146	147	1	0.00133	0.409	0.135
Branch model	*Cannabis-Humulus REM16* ancestor	42 genes 245 codons	two-ratio	−25,959.19	−25,954.63	9.126526	146	147	1	0.00252	0.403	0.314
Branch model	*Cannabis-Humulus-Arabidopsis REM16* ancestor	42 genes 245 codons	two-ratio	−25,957.71	−25,952.87	9.68	146	147	1	0.00185	0.409	0.09
Branch model	*REM16* sub-clade (6 tips + internals)	42 genes 245 codons	two-ratio	−25,954.90	−25,940.68	28.442778	146	147	1	9.65E-08	0.429	0.185
Branch-site model A	*Cannabis-Humulus REM16* ancestor	42 genes 245 codons	branch-site	−25,740.33	−25,739.95	0.747654	148	149	1	ns (0.3872)	NA	NA

Focusing then on our candidate sex-determination gene, a two-ratio branch model was fitted on the *REM16* clade, with the *CsREM16* (LOC115699937) terminal branch tagged as foreground; this yielded ω = 0.094, significantly lower than the background ω of 0.411 for the rest of the clade (LRT = 16.77, df = 1, p = 4.2 × 10^−5^; **Table 3**). This indicates that *CsREM16* is under strong purifying selection. The same model, run for its close ortholog in *H. lupulus*, *HlREM16*, found it likewise under strong purifying selection, albeit at a higher level than *CsREM16* at ω = 0.135 (LRT = 10.3, df = 1, p = 0.00133; **Table 3**).

The branch ancestral to the *Cannabis–Humulus REM16* orthologs also showed significantly lower ω (0.314) compared to the neutral expectation (LRT = 9.13, df = 1, p = 2.5 × 10^−3^; background ω = 0.403; **Table 3**). To further analyze whether there are specific amino acid residues under positive selection, a branch-site model test on the *Cannabis–Humulus REM16* ancestral branch was conducted. This yielded close to identical log-likelihoods for the null and alternative hypotheses (**Table 3**), providing no evidence for positive selection at individual sites along this branch. Lastly, a two-ratio branch model was also fitted on the ancestor preceding the *Cannabis–Humulus–Arabidopsis REM16* clade, again recovering a significantly lower foreground ω (0.09) relative to the background ω of 0.409 (LRT = 9.68, df = 1, p = 1.85 × 10^−3^; **Table 3**), suggesting that the extremely strong purifying selection on *REM16* predates even the divergence of *A. thaliana* from the *Cannabis–Humulus* lineage.

### 3.4 Differential expression analysis

To investigate whether B3-domain transcription factor genes exhibit sex-biased expression in *C. sativa*, differential gene expression analysis between male and female samples was performed independently at six developmental stages in the *C. sativa* cultivar ‘FINOLA’: germination, cotyledon, first leaf (L1), second leaf (L2), fourth leaf (L4), and ninth leaf pair (L9).

Each developmental stage was defined based on distinct morphological criteria, and specific tissues were collected for RNA sequencing accordingly. The germination stage was characterized by radicle emergence while the seed coat remained attached. For this stage, the naked seeds (without seed coat) excluding the radicle were used for RNAseq (**Figure 4A**). At the cotyledon stage, seedlings displayed fully expanded cotyledons but had not yet developed visible true leaves. RNA was isolated from a single cotyledon including SAM (**Figure 4B**). The L1 plants were defined by the presence of a fully developed first pair of true leaves, with the second pair not exceeding 5 mm in length. Apical meristem tissue was collected at this stage (**Figure 4C**). For the later developmental stages (L2, L4, and L9), we leveraged previously published RNAseq datasets (Shi et al., 2025) in which tissue was collected from the apical meristem. The L2 stage is vegetative in both male and female individuals, while L4 is already flowering in males but is vegetative in females, and L9 is flowering in females.

**Figure 4:**
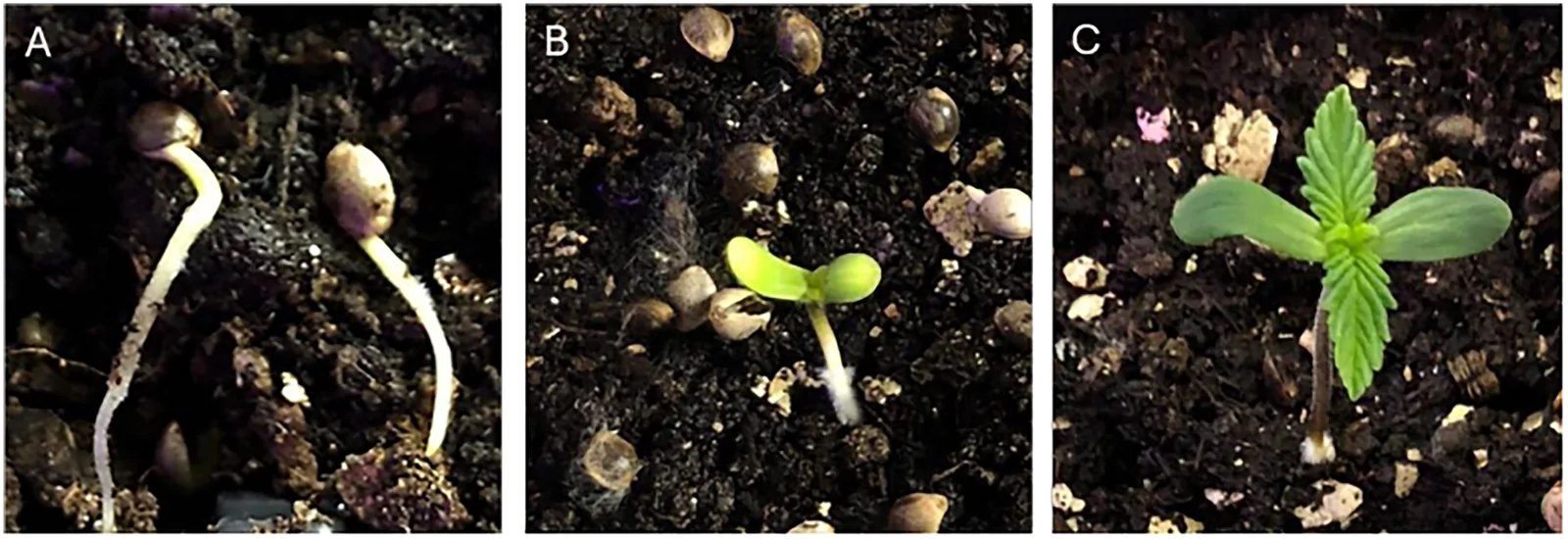
Representative images of the developmental stages used in the study. **(A)** Germination stage, **(B)** cotyledon stage, and **(C)** L1 stage. Developmental stages were defined according to the morphological criteria described in the main text and used for tissue collection prior to RNA sequencing.

RNAseq data generated in this study for the germination, cotyledon, and L1 stages were integrated with the published datasets for L2, L4, and L9 to enable a comprehensive analysis of sex-biased gene expression across a developmental progression.

For each developmental stage comparison, genome-wide differential expression was assessed, and results were subsequently filtered to retain only members of the B3 gene family (**Supplementary Table S3**). As male plants flower at L4, while female plants flower only at L9 (Shi et al., 2025), we also included a comparison of L4 male (L4M) vs. L9 female (L9F) samples.

Of the 100 annotated B3 genes in the *C. sativa* genome, 26 displayed either none or consistently very low expression (failed to meet the minimum expression threshold of 10 reads in at least two samples) across samples (**Supplementary Table S4**). The majority of these genes (23 out of 26) belonged to the REM family, while the remaining three were classified as RAV, ARF, and ABI3, respectively. This indicates that approximately 11% of non-REM genes (3 out of 28) show little to no expression, compared with nearly 32% of REM genes (23 out of 72) exhibiting low or no expression. Notably, 13 of the 23 lowly or non-expressed REM genes are located in the cluster of REM genes on chromosome 4 that consists of 36 genes, i.e., 36.1% of the genes on the chromosome 4 cluster are not expressed. Of the remaining 36 *C. sativa* REM genes, a slightly lower proportion 10 out of 36 (27.8%) are not expressed in our dataset.

Genes with no or very low expression were excluded from subsequent analyses, and the remaining 74 expressed genes were retained for stage-specific differential expression analysis. Among the 74 expressed B3 genes, 22 (almost 30%) showed significant sex-biased expression (FDR <0.05) in at least one developmental stage comparison (**Figure 5**). Notably, 18 of these genes belong to the REM subfamily (25% of the total REM genes) (**Figure 5A**), while only two were ARF (12% of the total ARF), one RAV (20% of the total RAV), and one HSI (33% of the total HSI). Comparing REM genes vs. non-REM B3 genes indicated a strong enrichment of sex-biased expression within the REM lineage (Fisher’s exact test p-value = 0.04461), with approximately three times higher odds of showing sex-biased expression compared to non-REM B3 genes (OR = 3.048). The cotyledon stage displayed the highest number of sex-biased B3 genes (**Figure 5B**).

**Figure 5:**
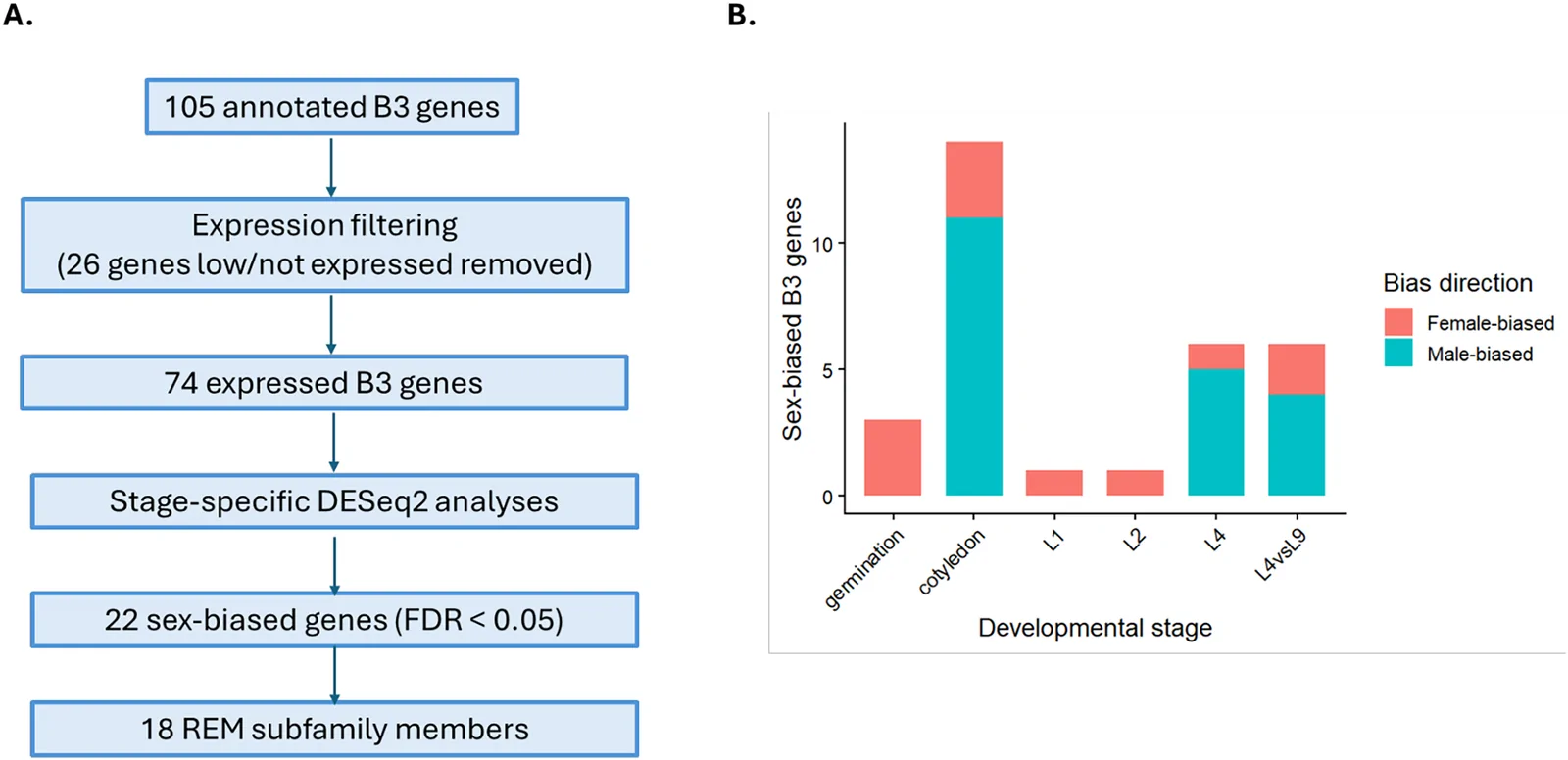
Identification of sex-biased B3 genes across development. **(A)** Workflow summarizing filtering and stage-specific differential expression analysis used to identify REM-enriched sex-biased B3 genes. **(B)** Number of B3 genes showing significant sex-biased expression at each developmental stage, colored by direction of bias.

Only one B3 gene, *CsREM16*, exhibited consistent sex-biased expression across all six developmental stage comparisons, with a uniform direction (female bias) throughout development (**Figure 6**; **Table 4**). In contrast, *CsREM10* (LOC115697055, chromosome 7) was differentially expressed in three developmental stage comparisons (cotyledon, L4, and L4MvsL9F), but the direction of bias varied across development, being female-biased at the cotyledon stage and male-biased at later stages (**Figure 6B**; **Table 4**).

**Figure 6:**
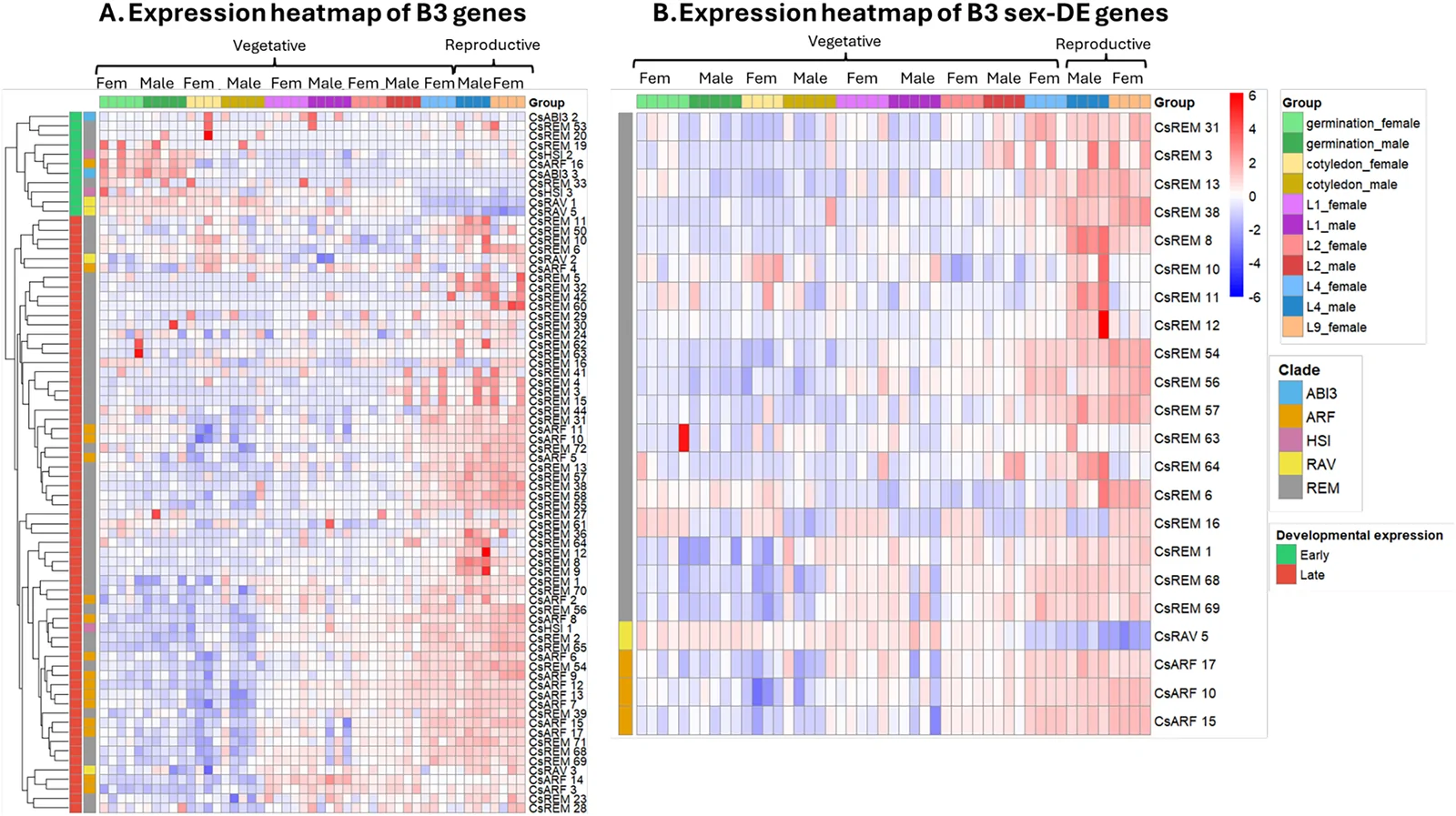
Expression profiles of B3 domain genes in *C. sativa*. Heatmaps of rlog-transformed, row-scaled (z-score) expression values across six developmental stages in male and female plants. Columns represent individual samples grouped by stage and sex. **(A)** A total of 74 B3 genes expressed above the threshold (>10 counts in >3 samples). **(B)** A total of 22 genes with significant sex-biased expression in at least one stage. Colors indicate relative expression (red = higher, blue = lower).

**Table 4:** Sex-differentially expressed B3 genes in *Cannabis sativa*.

Gene ID	Chromosome	Gene name	Subfamily	Stage and bias
LOC115699937	X	*CsREM16*	REM	All: female
LOC115697055	7	*CsREM 10*	REM	Cotyledon: female
				L4: male
				L4vsL9: male
LOC115697059	7	*CsREM12*	REM	L4: male
				L4vsL9: male
LOC115697158	7	*CsREM11*	REM	L4: male
				L4vsL9: male
LOC115697054	7	*CsREM8*	REM	L4: male
LOC115701709	X	*CsARF15*	ARF	Cotyledon: male
LOC115703019	X	*CsARF17*	ARF	Cotyledon: male
LOC115703033	X	*CsREM13*	REM	Cotyledon: male
LOC115712049	4	*CsREM6*	REM	L4: male
LOC115712352	4	*CsREM31*	REM	Cotyledon: male
LOC115712608	4	*CsREM57*	REM	Cotyledon: female
LOC115712999	4	*CsREM54*	REM	Cotyledon: male
LOC115713000	4	*CsREM56*	REM	L4vsL9: female
LOC115713776	4	*CsREM38*	REM	Cotyledon: male
LOC115714716	4	*CsREM3*	REM	Cotyledon: male
LOC115716126	5	*CsARF10*	ARF	Cotyledon: male
LOC115717024	5	*CsREM64*	REM	Germination: female
LOC115717029	5	*CsREM63*	REM	Germination: female
LOC115723020	9	*CsREM1*	REM	Cotyledon: male
LOC115724716	6	*CsREM68*	REM	Cotyledon: male
LOC115725443	6	*CsRAV5*	RAV	L4vsL9: male
LOC133032423	X	*CsREM69*	REM	Cotyledon: male

Two genes, *CsREM11* and *CsREM12*, also located on chromosome 7, showed male-biased expression specifically in comparisons in flowering stages (L4 and L4MvsL9F).

The remaining differentially expressed genes showed stage-specific sex bias: 12 were significant exclusively at the cotyledon stage, two at germination, two only in the L4 comparison, and two at the L4MvsL9F comparison (**Figure 6B**; **Table 4**).

Independent of plant sex, analysis of normalized expression patterns across all B3 genes revealed a general trend of increased expression at later developmental stages, as 85% of the expressed genes showed this pattern (**Figure 6A**). While sex-biased expression is restricted to a subset of B3 genes (**Figure 6B**), upregulation later in development, particularly in reproductive development, seems to be a broader feature of this transcription factor family.

Using publicly available RNAseq data from leaves of mature flowering plants (Prentout et al., 2021), we further examined the expression of B3 genes in hops, with particular focus on the REM subfamily. Hops contain a total of 88 B3 genes, of which only 50 showed detectable expression in the available RNAseq data; 25 of these belonged to the REM subfamily (**Figure 7A**). Overall, 11 genes were found to be differentially expressed between male and female samples (**Figure 7B**), including five members of the REM group (**Table 5**). Among these 11 genes, four have orthologous relationships with sex-biased genes previously reported in *C. sativa*: specifically, *HlREM52* corresponds to *CsREM69*, *HlREM16* to *CsREM16*, *HlRAV5* to *CsRAV5*, and *HlREM12* to *CsREM10*.

**Figure 7:**
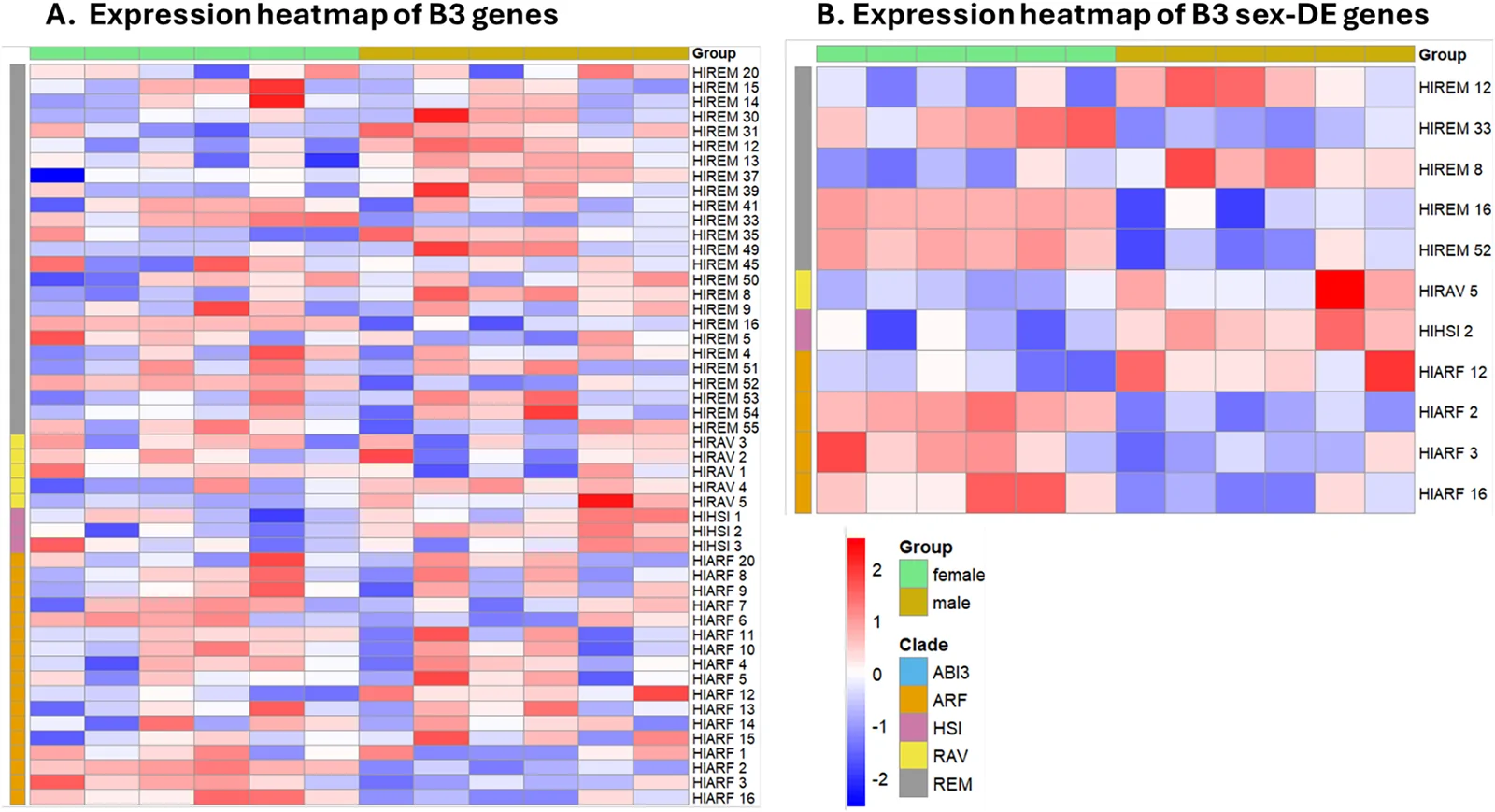
Expression profiles of B3 domain genes in *H. lupulus*. Heatmaps of rlog-transformed, row-scaled (z-score) expression values in male and female leaves in hops. Columns represent individual samples grouped by sex. **(A)** A total of 53 B3 genes expressed above the threshold (>10 counts in >3 samples). **(B)** A total of 11 genes with significant sex-biased expression. Colors indicate relative expression (red = higher, blue = lower).

**Table 5:** Sex-differentially expressed B3 genes in *Humulus lupulus*.

Gene ID	Sex-bias	Chromosome	Gene name	Subfamily
LOC133788586	Female	7	*HlARF3*	ARF
LOC133798586	Female	8	*HlARF2*	ARF
LOC133797716	Female	1	*HlREM52*	REM
LOC133803719	Female	X	*HlREM16*	REM
LOC133788585	Female	7	*HlARF16*	ARF
LOC133811830	Female	1	*HlREM33*	REM
LOC133800996	Male	9	*HlREM12*	REM
LOC133830142	Male	4	*HlREM8*	REM
LOC133797335	Male	8	*HlARF12*	ARF
LOC133782923	Male	6	*HlRAV5*	RAV
LOC133800559	Male	9	*HlHSI2*	HSI

### 3.5 Expression of *CsREM16* and its orthologs

Our data indicate that *CsREM16* is female-biased in expression in the *C. sativa* cultivar ‘FINOLA.’ We conducted qPCR analysis to determine whether this pattern is conserved in other hemp cultivars, including ‘Kompolti,’ a dioecious cultivar of different origin, and ‘Felina 32,’ a monoecious cultivar. The results showed that *CsREM16* is consistently overexpressed in XX plants compared to XY, with overexpression detected from very early stages. Expression levels are similar across cultivars (**Figures 6B**, **8A**).

**Figure 8:**
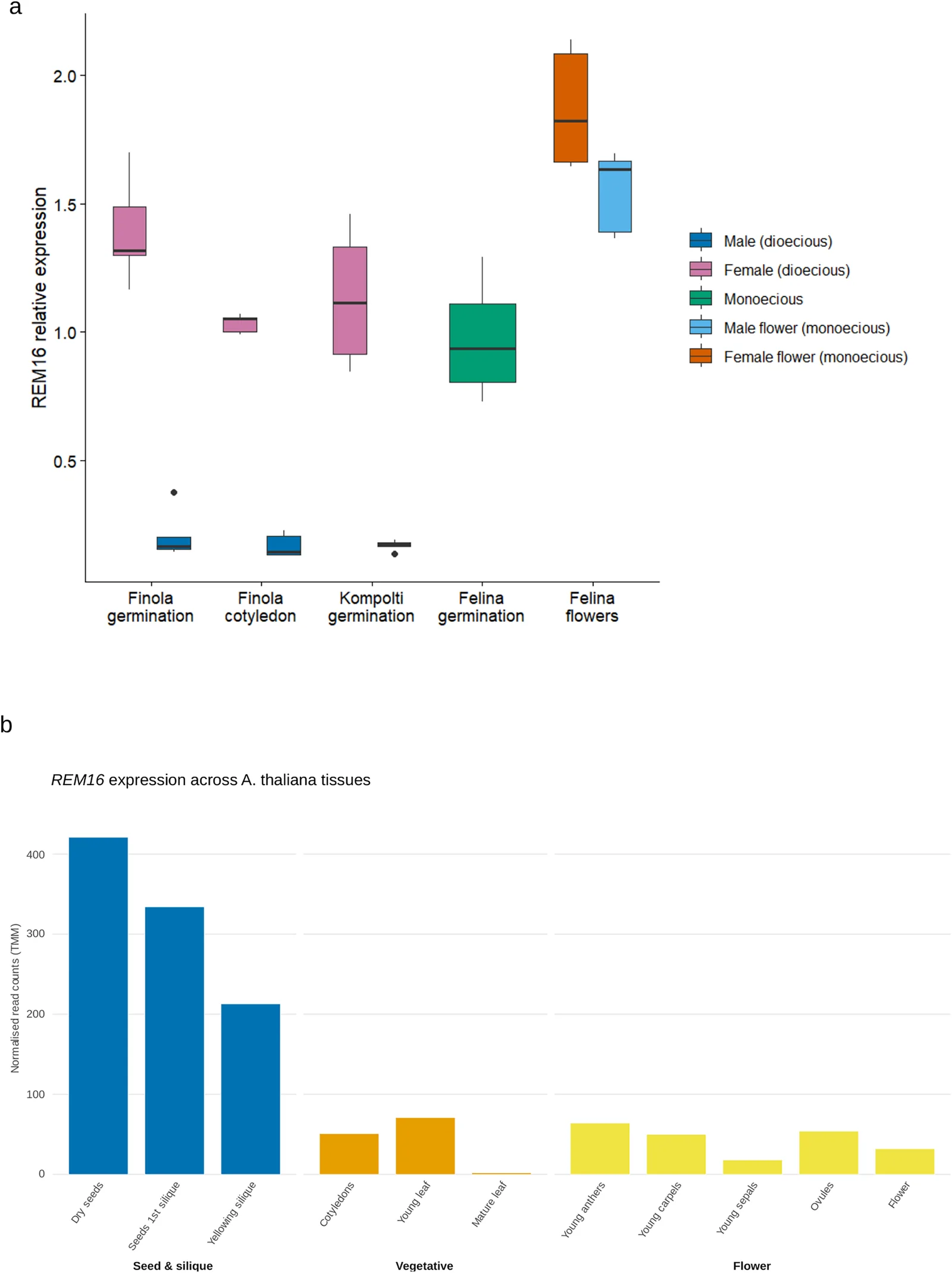
Expression of *REM16* across *C. sativa* cultivars and *A. thaliana* tissues. **(A)** Relative expression of *CsREM16* in XX and XY plants across three hemp cultivars (‘FINOLA,’ ‘Kompolti,’ and ‘Felina 32’) at early developmental stages, measured by qPCR. *CsREM16* is consistently overexpressed in XX plants compared to XY across all cultivars. **(B)** Expression of *REM16* across developmental stages and tissues in *A. thaliana*, based on publicly available data. *REM16* is strongly upregulated in seeds relative to other tissues and developmental stages.

The data from hops further show that *HlREM16* is also female-biased in its expression (**Figure 7B**) (Prentout et al., 2021; Toscani et al., 2026). We next used publicly available data from *A. thaliana* to analyze *REM16* expression in different tissues (Klepikova et al., 2016). This indicates that *REM16* is strongly upregulated in embryos but expressed at low levels, if at all, in other developmental stages (**Figure 8B**).

## 4 Discussion

B3 domain proteins are key regulators of plant development. Here, we identified a total of 299 putative B3 domain-containing genes across *A. thaliana* (110), *C. sativa* (101), and *H. lupulus* (88), resolving them after phylogenetic reconstruction into the five canonical subfamilies: *ARF*, *ABI3*, *HSI*, *RAV*, and *REM*. The gene counts we recovered for *A. thaliana* exceeded the 87 reported in the foundational Romanel et al. (2009) study and the 92 identified more recently (Peng and Weselake, 2013), likely reflecting improvements in genome annotation and the higher sensitivity of the multi-strategy GENE-FAM pipeline used here (Ryan et al., 2026), which combines protein- and nucleotide-level searches alongside whole-genome scanning. Subfamily composition in *A. thaliana* was largely consistent with what was described previously, with ABI3 (3), HSI (3), RAV (13), and ARF (19) counts corresponding to Romanel et al. (2009), while the REM family gene count of 72 was higher than the 45 reported. For *C. sativa*, the only prior genome-wide survey identified 65 B3 members (Lu et al., 2024), making our identification of 101 genes a considerable expansion. This is likely attributable both to differences in the newer Pink Pepper (GCF_029168945.1) reference genome assembly used compared to the previous CBDRx (GCF_900626175.2), and to the GENE-FAM pipeline. In all three species, the REM subfamily was by far the largest B3 subfamily, consistent with figures previously reported in other species (Park et al., 2024; Wang et al., 2024).

For some REM genes, like *CsREM16* and *VRN1*, clear orthologs were identifiable across all three species, indicative of a high degree of conservation over large evolutionary timescales. The *REM16* clade also exhibited consistently low ω values across its members, consistent with strong purifying selection acting across the clade. Notably, *CsREM16* itself displayed a remarkably low ω value of 0.09, a value that coincides strikingly with that reported for MeGI (Akagi et al., 2020), the sex-determining gene in persimmon, suggesting that high functional constraint on sex-determining genes may be a conserved feature across distantly related dioecious species. The absence of detectable positive selection on the branches leading to *CsREM16* suggests that, if neofunctionalization is responsible for its acquisition of sex-determination ability, the responsible changes may not be encoded in the protein sequence itself, or at least not in the domains analyzed here, but rather in a change in expression patterns. Indeed, in *A. thaliana*, *REM16* is highly expressed early in seed development but expressed at low levels, if at all, later in development (**Figure 8**). This is in stark contrast to *C. sativa*, where *CsREM16* is expressed at all stages of plant development but exclusively in female or monoecious (i.e., XX) and not in male plants. We speculate that a change in expression in the lineage leading to *CsREM16* and *HlREM16* facilitated their recruitment as sex-determination genes in *C. sativa* and *H. lupulus*.

The analysis of normalized expression patterns across a broad range of developmental stages in *C. sativa* indicates that the general trend for B3 genes is increased expression at later developmental stages, suggesting that a role in reproductive development is a main function of genes in this family, similar to what has been proposed for *A. thaliana* (Mantegazza et al., 2014). In contrast, assessment of sex-biased expression across development showed that such bias is limited to a subset of genes and is highly dependent on the developmental stage. Approximately 30% of the expressed B3 genes showed significant sex-biased expression in at least one comparison; however, most of the genes were not consistently sex-biased across multiple developmental stages. The sex-biased genes had a significant enrichment in the REM group, and it remains to be seen whether REM genes in general are involved specifically in the development of male or female plants.

The phylogenetic reconstruction of the B3 superfamily revealed a striking contrast between the evolutionary trajectories of different B3 subfamilies. While the ARF, ABI3, HSI, and RAV clades displayed phylogenetic relationships broadly congruent with species relationships, with often clear orthologs identifiable across all three species, the REM clade was dominated by large species-specific clades and *C. sativa–H. lupulus*-only clades, making unambiguous cross-species orthology assignments frequently impossible. This pattern is consistent with a history of recurrent, lineage-specific gene duplications and reflects observations in other plant lineages where the REM family has been reported as the most dynamically evolving B3 subfamily (Park et al., 2024; Zameer et al., 2025).

Patterns consistent with tandem duplication were particularly prominent within the REM clade. Across all three species, REM genes were far more likely to occur within 100 kb of another B3 family member than non-REM B3 genes (64.3% vs. 14.1%), and this tendency was most pronounced in *C. sativa*, where 79.2% of REM genes were found in close genomic proximity to one another. The most extreme example is a dense cluster of 36 REM genes spanning only approximately 350 kb on *C. sativa* chromosome 4, the large majority of which are located on the same forward strand, a hallmark of tandem duplication. A comparable, though smaller, cluster of 19 REM genes on *H. lupulus* chromosome 1 shows a similar strand bias (95% forward strand). Phylogenetic analysis of the chromosome 4 cluster genes revealed that several have their closest relatives in the *H. lupulus* chromosome 1 cluster, indicating that the ancestral tandem array predates the *Cannabis–Humulus* divergence, while the two clusters continued to expand independently after speciation. This pattern is accompanied by a higher ratio of nonsynonymous to synonymous substitutions (ω) for these genes. Furthermore, more than one-third of the REM genes on the chromosome 4 cluster in *C. sativa* were expressed at very low levels, if at all. Together, the picture emerges that REM genes undergo bursts of tandem duplications, leading to gene non-functionalization but potentially also sub- and neofunctionalization. Some well-characterized *Arabidopsis* genes, like *VAL* or *VDD*, also seem to be part of recent duplication bursts (**Figures 2**, **3A**), and it will be interesting to see whether those genes function partially redundantly with closely related paralogs.

## Data Availability

The newly generated RNA-seq data has been uploaded to the NCBI SRA archive under bioproject PRJNA1540498.
