# Supplementary material for: Rapid bursts and long-term stasis: phylogeny, evolutionary dynamics, and expression of B3 transcription factors in Arabidopsis, Cannabis, and Humulus
Source: Front Plant Sci. 2026 Oct 15;17:1882604. doi: 10.3389/fpls.2026.1882604 (PMC13717154; doi:10.3389/fpls.2026.1882604)
Supplement: Supplementary file 1 [file DataSheet1.pdf]

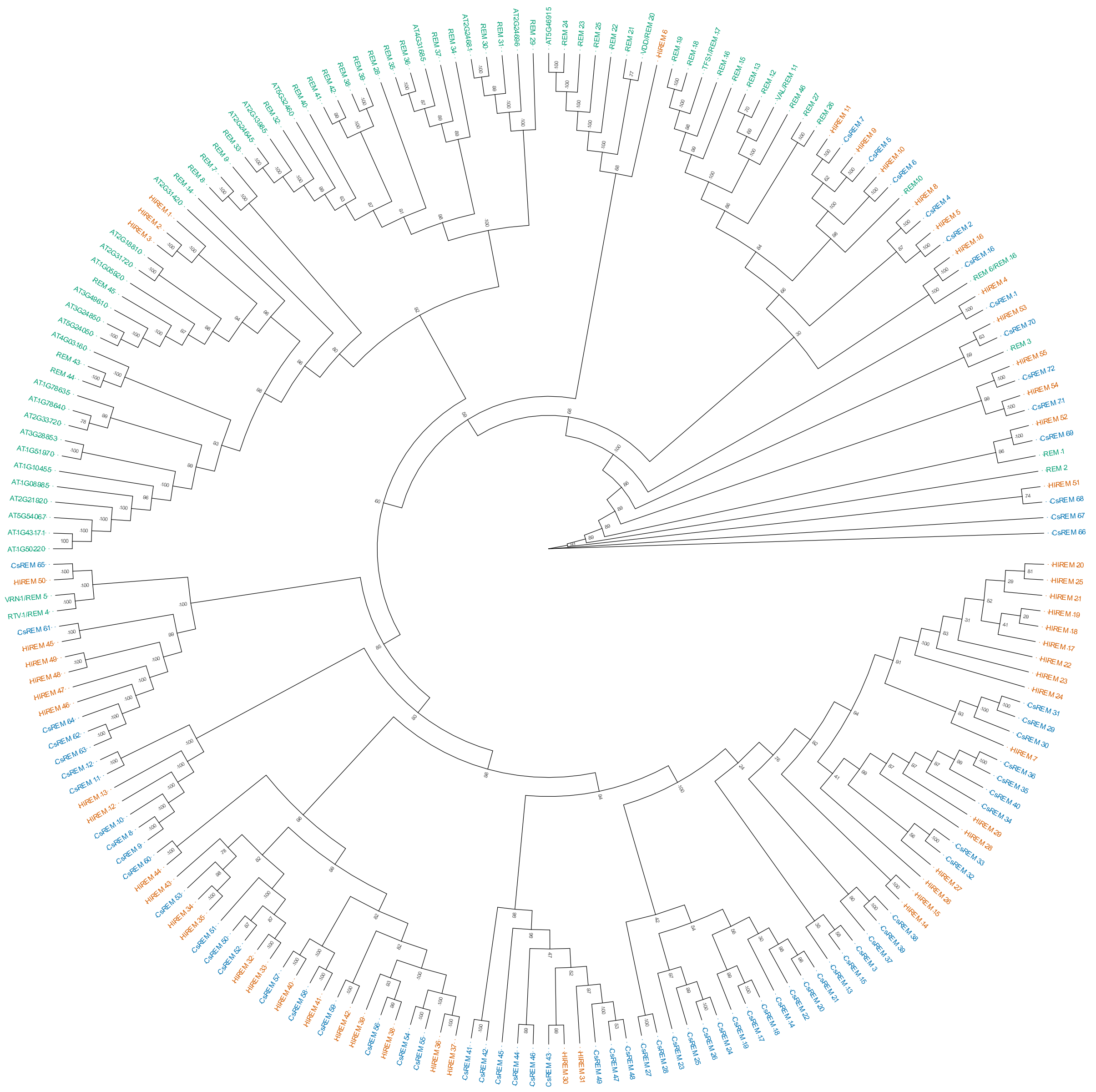

Figure S1. Detailed phylogenetic reconstruction of the REM gene subfamily.
Maximum-likelihood phylogenetic tree specifically focusing on the REM (Reproductive Meristem) family clade genes from *Arabidopsis thaliana*, *Cannabis sativa*, and *Humulus lupulus*. The tree is visualized in a circular layout with branch lengths omitted to clearly display topological relationships. Tip labels denote individual gene names and are connected to the tree via dotted leader lines. Labels are color-coded according to species of origin: *A. thaliana* (bluish-green), *C. sativa* (dark blue), and *H. lupulus* (vermillion). Numbers located at the internal nodes (dark grey) indicate bootstrap support values from the maximum-likelihood analysis.

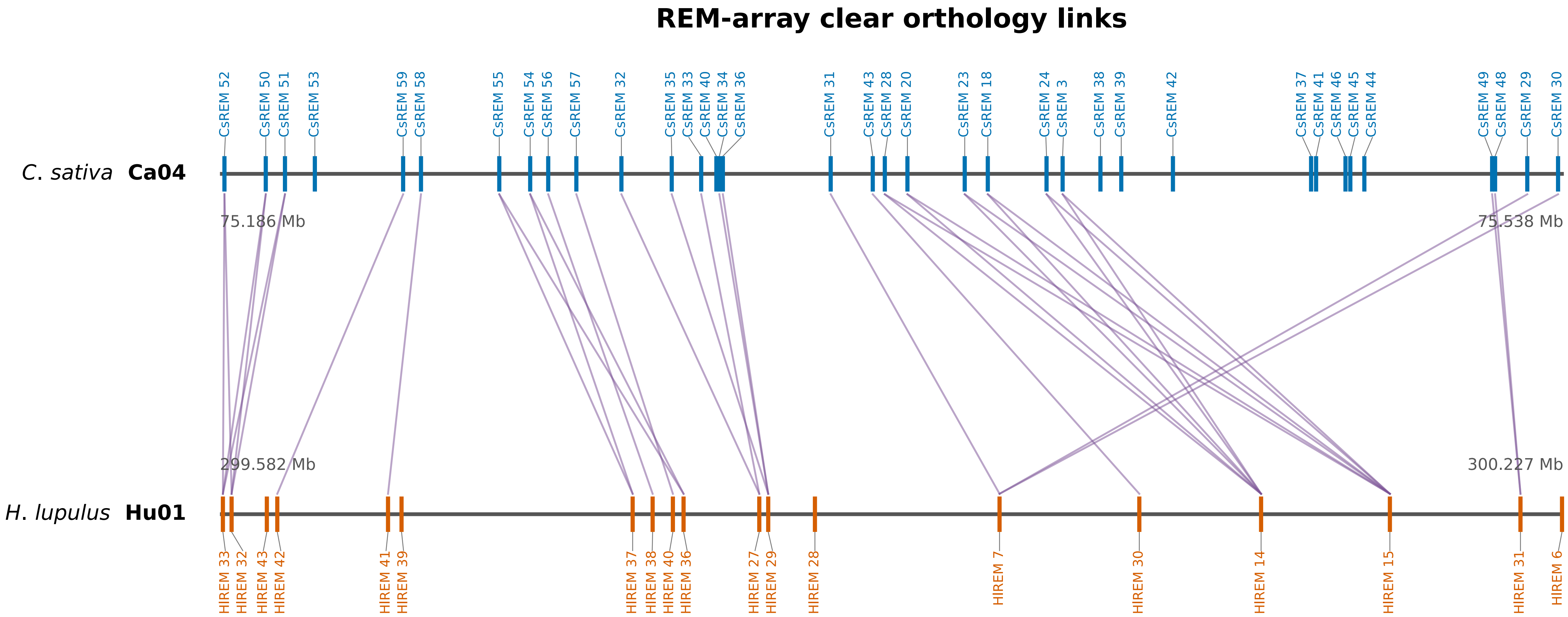

Figure S2. Collinearity on phylogeny-based orthology relationships of the *Cannabis sativa* and *Humulus lupulus* REM arrays.

The 36 *REM* genes in the *C. sativa* chromosome 4 array (Ca04; top, blue) and the 19 *REM* genes in the *H. lupulus* chromosome 1 array (Hu01; bottom, orange) are shown in genomic order. Purple lines connect genes whose most recent common ancestor in the phylogeny was a speciation node (clear othology relation). Only relationships between genes in the two displayed tandem arrays are drawn. Multiple links from one gene indicate co-orthology relationships arising from lineage-specific duplication. No clear *C. sativa* ortholog was assigned to *HlREM28*, *HlREM39*, *HlREM43*, or *HlREM6* under this criterion.

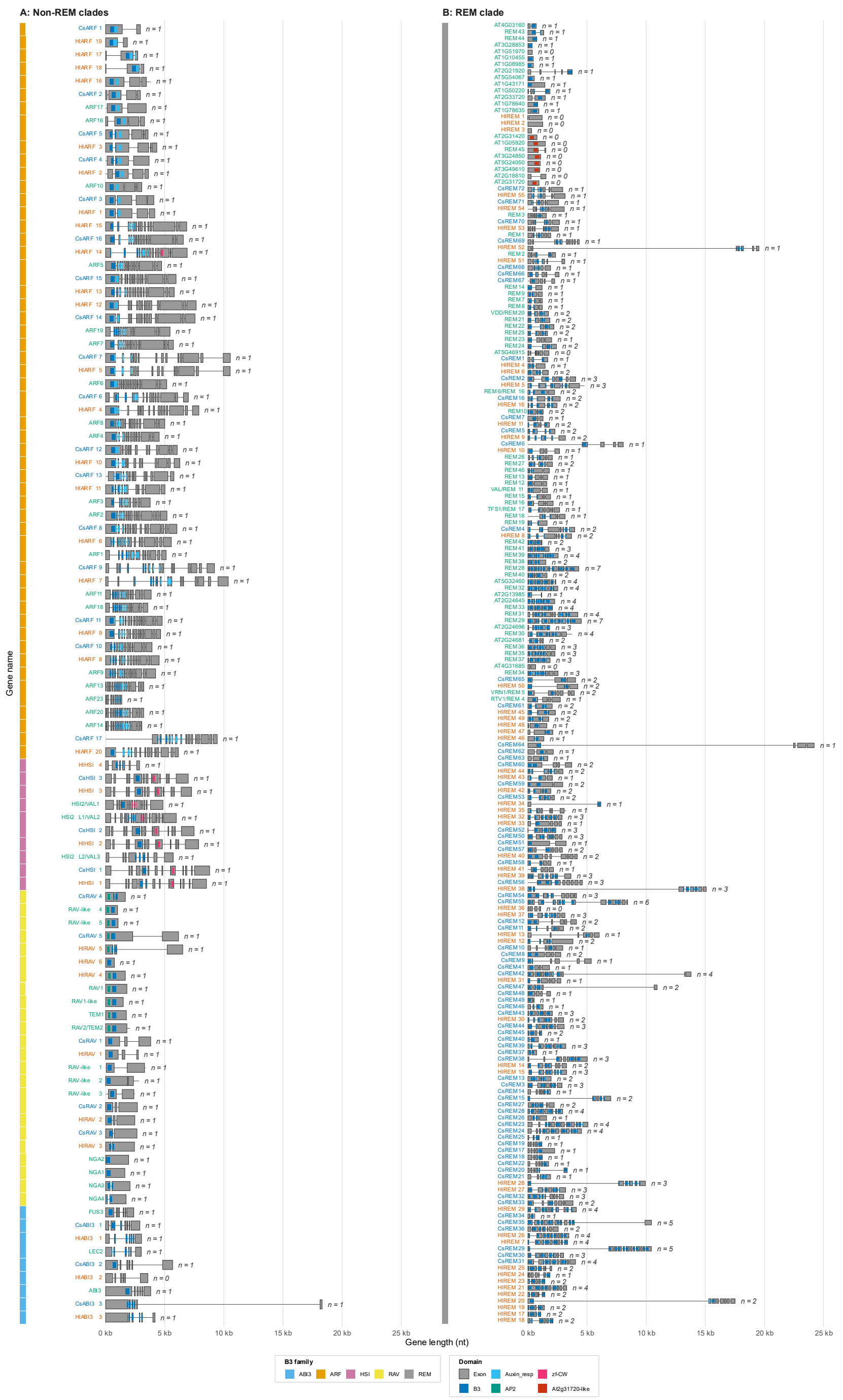

Figure S3. Gene structure and conserved protein domain position architecture of the B3 gene superfamily.
Intron-exon organization and the distribution of conserved protein domains across the identified B3 genes in *Arabidopsis thaliana*, *Cannabis sativa*, and *Humulus lupulus*. Grey boxes represent exons while the connecting lines indicate introns. Conserved domains, identified via the NCBI Conserved Domain Database (CDD), are overlaid as colored blocks: the defining B3 domain (blue), Auxin_resp (cyan), AP2 (orange), ZnF_CW (red), and zf-B_box (magenta). Gene identifiers on the y-axis are color-coded by species and the vertical strip denotes the B3 subfamily clade for each gene assigned in the phylogeny. It shows distinct clade-specific patterns with the ARF and HSI families harbing more fragmented gene structures (median of 13 exons each), whereas the RAV clade genes possess often few to no introns (median of 2 exons). To the right of each gene the B3 domain count is shown. Notably, while most clades typically contain a single B3 domain per gene, the REM clade is distinguished by the frequent presence of multiple B3 domains (most frequently 2 domains per gene).
